# Transcutaneous auricular vagus nerve stimulation can modulate fronto-parietal brain networks

**DOI:** 10.3389/fnins.2024.1368754

**Published:** 2024-07-18

**Authors:** Sang-Yoon Han, Leeseul Shim, Hyo-Jeong Lee, Moo Kyun Park

**Affiliations:** ^1^Department of Otolaryngology-Head and Neck Surgery, College of Medicine, Hanyang University, Seoul, Republic of Korea; ^2^Laboratory of Brain and Cognitive Sciences for Convergence Medicine, Hallym University College of Medicine, Anyang-si, Republic of Korea; ^3^Ear and Interaction Center, Doheun Institute for Digital Innovation in Medicine, Hallym University Medical Center, Anyang-si, Republic of Korea; ^4^Department of Otorhinolaryngology-Head and Neck Surgery, Hallym University College of Medicine, Chuncheon-si, Republic of Korea; ^5^Department of Otorhinolaryngology-Head and Neck Surgery, Seoul National University Hospital, Seoul, Republic of Korea; ^6^Sensory Organ Research Institute, Seoul National University, Medical Research Center, Seoul, Republic of Korea

**Keywords:** transcutaneous vagal nerve stimulation, brain mapping, fronto-parietal network, neurostimulation, neuromodulation

## Abstract

**Objective:**

Recent studies have shown that transcutaneous vagal nerve stimulation (tVNS) holds promise as a treatment for neurological or psychiatric disease through the ability to modulate neural activity in some brain regions without an invasive procedure. The objective of this study was to identify the neural correlates underlying the effects of tVNS.

**Methods:**

Twenty right-handed healthy subjects with normal hearing participated in this study. An auricle-applied tVNS device (Soricle, Neurive Co., Ltd., Gyeongsangnam-do, Republic of Korea) was used to administer tVNS stimulation. A session consisted of 14 blocks, including 7 blocks of tVNS stimulation or sham stimulation and 7 blocks of rest, and lasted approximately 7 min (1 block = 30 s). Functional magnetic resonance imaging (fMRI) was performed during the stimulation.

**Results:**

No activated regions were observed in the fMRI scans following both sham stimulation and tVNS after the first session. After the second session, tVNS activated two clusters of brain regions in the right frontal gyrus. A comparison of the activated regions after the second session of each stimulation revealed that the fMRI following tVNS exhibited four surviving clusters. Additionally, four clusters were activated in the overall stimulated area during both the first and second sessions. When comparing the fMRI results after each type of stimulation, the fMRI following tVNS showed four surviving clusters compared to the fMRI after sham stimulation.

**Conclusion:**

tVNS could stimulate some brain regions, including the fronto-parietal network. Stimulating these regions for treating neurological or psychiatric disease might require applying tVNS for at least 3.5 min.

## Introduction

Since some neurological and psychiatric disease such as epilepsy, depression, dementia, and tinnitus is influenced by the autonomic nervous system, interventions targeting this system have been explored (Howland, 2014; Yap et al., 2020; Kim et al., 2022; Vargas-Caballero et al., 2022; Patil et al., 2023). Vagus nerve stimulation (VNS) is one such intervention, which involves applying micro-currents to the vagus nerves through electrical stimulation (Ventureyra, 2000; Howland, 2014). VNS has been shown to effectively improve a variety of neurological conditions, including headaches, Alzheimer’s disease, sleep disorders, epilepsy, and tinnitus (Howland, 2014; Stegeman et al., 2021; Vargas-Caballero et al., 2022). However, VNS is an invasive procedure that requires cervical dissection and the surgical implantation of a device, which has limited its widespread use as a treatment option for these conditions (Lehtimäki et al., 2013; Vargas-Caballero et al., 2022). Additionally, the right vagus nerve predominantly innervates the sinoatrial (SA) node, while the left vagus nerve primarily targets the atrioventricular (AV) node. Consequently, based on the animal study, stimulation of the right vagus nerve can lead to cardiovascular issues such as bradycardia and asystole, potentially resulting in life-threatening situations (Chen et al., 2015; Yap et al., 2020). As a result, VNS is typically performed only on the left vagus nerve (Yap et al., 2020). To circumvent the risks associated with invasive VNS, transcutaneous VNS (tVNS) has been investigated.

Bilateral tVNS was administered to the cervical branch of the vagus nerve, located in the cavum conchae (Lehtimäki et al., 2013; Howland, 2014; Stegeman et al., 2021). Unlike conventional VNS, tVNS does not require an invasive surgical procedure and can be applied bilaterally with minimal risk of significant cardiac side effects (Chen et al., 2015; Yap et al., 2020). Based on speculation regarding the mechanisms of tVNS, the activation of the cervical vagus nerve by tVNS stimulates the epicardial ganglion plexus on both sides (Chen et al., 2015; Yap et al., 2020), avoiding direct cardiac stimulation and symmetrically stimulate the epicardial plexus, thus preventing asymmetric heart movements (Chen et al., 2015; Yap et al., 2020).

The signals generated by bilateral tVNS converge at the nucleus tractus solitarii, which then relays the signal to the caudal ventrolateral medulla and the dorsal motor nucleus (Chen et al., 2015; Yap et al., 2020), locus coeruelus which is thought to modulate synaptic plasticity by the release of catecholamines (Ventura-Bort et al., 2018; Berger et al., 2024), and the cholinergic nucleus basalis (Yakunina and Nam, 2021). This leads to changes of release of neuromodulator, influencing limbic, reticular, auditory, and autonomic centers of brain (Yakunina and Nam, 2021).

Additionally, many previous studies demonstrated that tVNS can suppress or activate specific brain region in healthy population or individuals with neurological disease (Yakunina et al., 2017, 2018; Naparstek et al., 2023). However, previous studies did not take into account factors such as sex, age, handedness, and the specific regions stimulated by tVNS (Yakunina et al., 2017, 2018; Stegeman et al., 2021; Naparstek et al., 2023).

The objective of this study was to identify the precise brain regions stimulated by tVNS. We employed tVNS and utilized functional magnetic resonance imaging (fMRI) to pinpoint the modulated regions. The study aimed to examine the brain regions affected by tVNS. We administered tVNS via the bilateral auricular branch and used fMRI to observe activations related to tVNS. To control for the somatosensory effects associated with electrical stimulation, sham stimulation was applied to the adjacent auricular skin.

To control for variations in brain activity patterns that could be attributed to aging or hand dominance, we exclusively recruited young to middle-aged subjects who were right-handed. Identifying the area stimulated by tVNS may be helpful in providing good reference values for brain activity change after tVNS.

## Materials and methods

### Subjects and study protocol

Twenty right-handed, healthy subjects with normal hearing participated in this study. Subjects were excluded if they had any history of neurological or neuropsychiatric diseases or were taking medications related to conditions such as diabetes and hypertension, as well as any conditions that contraindicated MRI scanning.

The recruited subjects visited the hospital once to participate in a functional brain imaging study utilizing tVNS. Each subject received a comprehensive explanation of the study’s purpose and procedures. They were also asked to complete a questionnaire designed to assess their health status, including physical condition and pain sensitivity.

Before entering the MRI scanner, all subjects were instructed to identify the optimal power level for their tVNS device. This was achieved by having each subject wear the tVNS device and incrementally adjust the stimulation intensity.

The same stimuli were used in both the active and the sham stimulation conditions, with 30 s of continuous stimulation at 30 Hz in each condition. The stimulation setup was the identical for both the active and sham conditions, the only difference between the two conditions was the location of the electrodes.

The process began at level 10, which is the maximum stimulation level (65 Vpp, 4.6 mA), and the intensity was reduced one level at a time until the subject felt most comfortable. Once the subject was positioned in the scanner, the power level previously set was reconfirmed to ensure it remained optimal. This precaution was taken to allow each subject to maintain their individual optimal power level throughout the duration of the fMRI experiment.

The experimenter asked participants directly about their condition before each scanning session to monitor their alertness and discomfort, and an external monitor was also used to monitor participants’ condition.

The average stimulation level utilized was 2.95 (SD = 1.05), with a range from level 1 (23 Vpp, 1.3 mA) to level 5 (45 Vpp, 3.1 mA).

The study protocol received approval from the Institutional Review Board of Hallym University Sacred Heart Hospital (Anyang, South Korea, IRB No. 2022-08-010-001). Written informed consent was obtained from each participant.

### Transcutaneous vagal nerve stimulation

**FIGURE 1:**
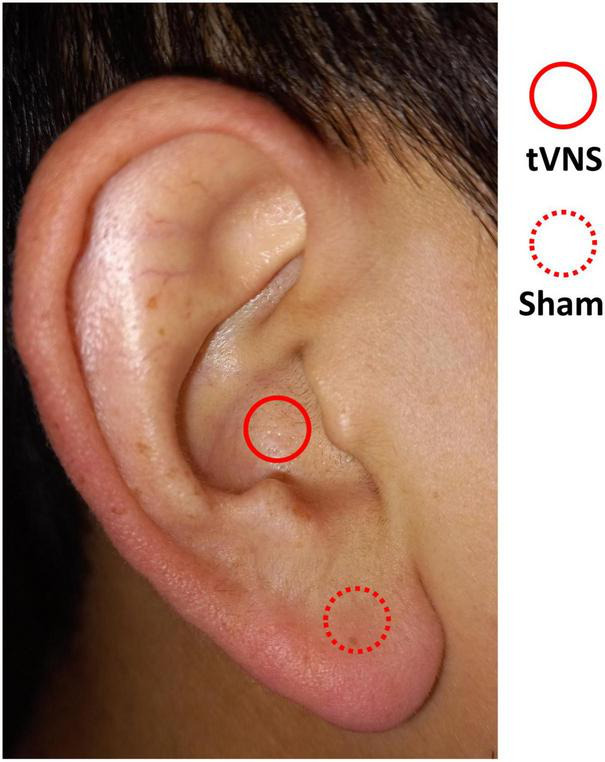
Areas of stimulation in the auricle. tVNS condition (solid line) and sham condition (dotted line).

In this study, we used Soricle (Neurive Co., Ltd., Gyeongsangnam-do, Republic of Korea), a device certified by the Korea Certification, to administer tVNS to the auditory branch of the vagus nerve. Bipolar electrodes were positioned on both sides of the auricle, with one pair on the cavum concha and another on the earlobe. Electrical stimulation was then applied to these two locations via the bilateral ears, delivering stimulation in accordance with the specified condition (Figure 1, tVNS: concha, sham: earlobe).

### fMRI experiment

Magnetic resonance images were acquired with a Siemens 3.0 T (Siemens Skyra, Erlangen, Germany). T1 MPRAGE images of the subject were obtained in multi-slice single-shot mode using the following parameters: repetition time (TR) = 2300 ms; echo time (TE) = 2.26 ms; flip angle = 8°; field of view (FOV) = 256 × 256 mm; matrix = 232 × 200; 416 slices; slice thickness = 0.5 mm with no gap. Functional magnetic resonance images were acquired using an interleaved multi-slice mode with the following settings: TR = 3000 ms; TE = 30 ms; flip angle = 90°; FOV = 90; and 75 slices with a thickness of 2 mm each.

To acquire (f)MRI images on Skyra, we used a multi-channel head coil containing head elements 1-4 (HE 1-4) and neck elements 1 and 2 (NE 1-2) and set the in-device coil selection mode to ‘Auto coil selection’.

The fMRI experiment utilized a block design with two distinct conditions: 1) the tVNS condition and 2) the sham condition. Each condition comprised two experimental sessions. During each session, which lasted approximately 7 min, participants were exposed to seven 30-s blocks of the stimulation condition (either tVNS or sham), interspersed with 30-s blocks of rest. This resulted in a total of 14 blocks per session. The tVNS and sham conditions were alternated, and the sequence of conditions was counterbalanced across participants. Subjects were blind to the stimulation condition throughout the experiment.

During the fMRI experiment, subjects were instructed to lie comfortably in the scanner with their eyes closed. The experimenter monitored the subjects’ level of attention and discomfort between sessions.

### fMRI data analysis

Imaging data were analyzed using the SPM 12 package (Wellcome Trust Centre for Neuroimaging, London, UK), which was implemented in MATLAB R2018b (MathWorks Inc., Natick, MA, USA). We performed standard preprocessing steps on both the functional and anatomical images, which included slice timing, motion correction, normalization, and smoothing. The functional images of the participants were co-registered with their respective anatomical T1 images. These images were then spatially normalized to a standard anatomical space, as defined by the MNI (Montreal Neurological Institute) T2 template image, by resampling them every 3 mm using sinc interpolation. Finally, the functional images were smoothed using a 6 mm full-width at half maximum (FWHM) Gaussian kernel.

In the first-level analysis, data from each participant were processed using a general linear model (GLM) with a single predictor that included four conditions: tVNS, rest during the tVNS session, sham, and rest during the sham session. The canonical hemodynamic response function (HRF) and a temporal high-pass filter with a cutoff of 128 s were applied to the predictors. Multiple regressors were employed as covariates to account for residual motion artifacts. Predictors corresponding to each experimental condition for each participant were then prepared for the second-level analysis.

In the second-level analysis, repeated-measures ANOVA was conducted, using each time point and condition as factors, with age included as a covariate of no interest, to compare the neural responses to tVNS and sham stimulation. Functional images from all sessions were entered in one GLM model of the second-level analysis. Then, areas of significant activation were searched in two sessions combined and each session separately using the same statistical threshold. The imaging data were initially thresholded at *p* = 0.0005 (uncorrected). However, only those clusters that achieved *p* = 0.05, corrected for family-wise error (FWE), were deemed significant and are reported.

## Results

### Demographic factors and underlying diseases of subjects

The mean age of the subjects was 33.65 ± 4.82 years, with an age range of 25–40 years. Of the subjects, five were men and 15 were women. One subject had been taking Synthroid, but none of the others had any underlying diseases.

### Associated symptoms and side effects of tVNS

There were no instances of skin burn or damage among the subjects. The pain visual analog scale for the tVNS procedure was 3.55 ± 1.99, with a range of 1 to 8. None of the subjects reported intolerable pain following tVNS. There were no significant differences in subjective fatigue, depressive mood, anxiety, or stress before and after tVNS, as shown in Table 1.

**TABLE 1:** Associated symptoms and possible side effects of transcutaneous vagal nerve stimulation before and after stimulation.

Symptoms (VAS 1∼8)	Pre-tVNS	Post-tVNS	*P*-value
Fatigue	4.20 ± 2.22	4.30 ± 2.45	0.602
Depressive mood	2.10 ± 1.52	2.05 ± 1.54	0.498
Anxiety	1.85 ± 1.14	1.95 ± 1.54	0.666
Stress	2.80 ± 1.80	2.65 ± 1.73	0.630

VAS, visual analog scale; tVNS, transcutaneous vagal nerve stimulation.

### Brain regions activated by tVNS in comparison to the sham stimulation

**FIGURE 2:**
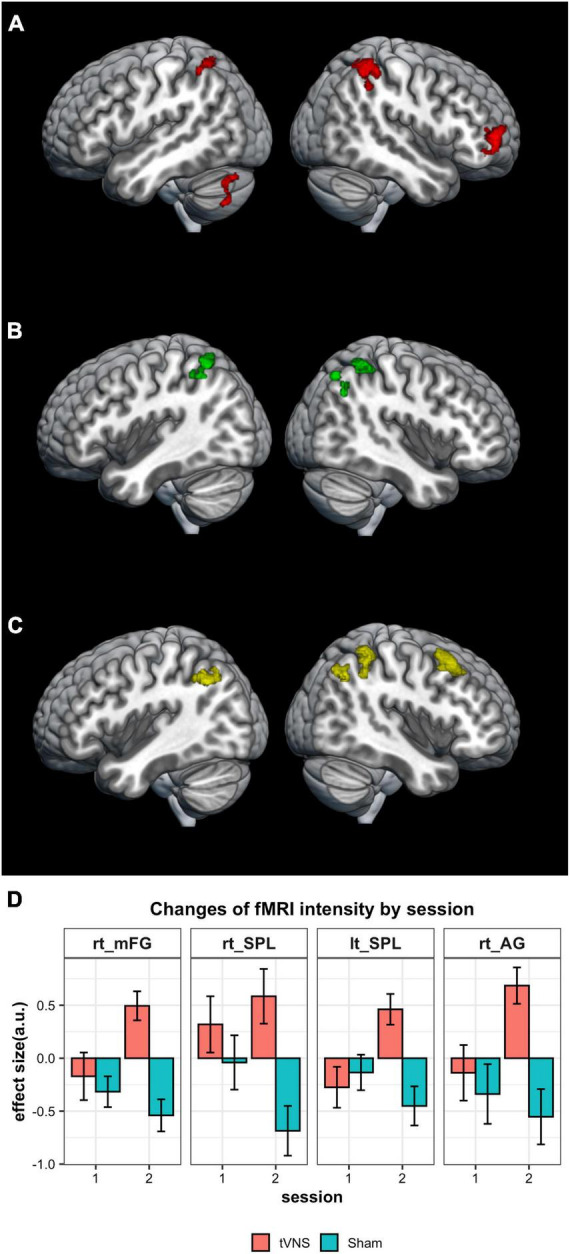
Results of imaging analysis. **(A)** Brain areas of significant activation for tVNS stimulation. **(B)** Regions of increased activation for tVNS condition over the sham condition are found in the fronto-parietal network. **(C)** Increased activation for tVNS was not observed in the first session, but was noted in the second session. **(D)** Changes of the response intensity by stimulation conditions in four regions shown in Figure 2C (a.u., arbitrary unit). The results are thresholded at *P* = 0.05 (FWE-corrected) at the cluster level.

We evaluated the stimulated regions over two sessions, one involving tVNS and the other sham stimulation. During tVNS, activation was observed in four clusters: the right middle frontal gyrus (40, 56, 2), the left superior parietal gyrus (−32, −58, 64), the right superior parietal gyrus (44, −44, 64), and the left cerebellum (−34, −70, −48) as shown in Figure 2A and Table 2. In contrast, sham stimulation did not result in any significant clusters of activation.

**TABLE 2:** Regions of significant neural activation in response to the tVNS and sham stimulation.

Regions	MNI coordinate			k	T	*P*-value (FWE-corrected)
	***x***	***y***	***z***			
**tVNS stimulation (concha)**						
Frontal pole, right	40	56	2	261	6.31	<0.001
Superior parietal lobule, left	−32	−58	64	114	5.27	0.036
Superior parietal lobule, right	44	−44	64	308	4.84	<0.001
Cerebellum, left	−34	−70	−48	111	4.40	0.039
**Sham stimulation (earlobe)**						
None						

MNI, Montreal Neurological Institute; FEW, family-wise error; tVNS, transcutaneous vagal nerve stimulation.

When comparing conditions, significantly higher activation was observed in the tVNS condition compared to the sham condition within three clusters: the left superior parietal gyrus (−32, −58, 64), the right precuneus (12, −68, 52), and the right superior parietal gyrus (48, −40, 54) (Figure 2B and Table 3). This significant difference between conditions was not observed in the first session but was replicated in the second session (Figures 2C, D and Table 3). In the second session, significantly higher activation was observed in the tVNS condition compared to the sham condition within four clusters: the right middle frontal gyrus (32, 20, 50), the right superior parietal gyrus (40, −46, 44), the left superior parietal gyrus (−34, −62, 40), and the right angular gyrus (44, −60, 60) (Figures 2C, D and Table 3). When applying the lower thresholds, areas in the contralateral hemisphere survived. A cluster in the left mFG appears at uncorrected *P* = 0.001 (mni coordinate = −38, 44, 6, T = 3.72) and in the right cerebellum at uncorrected *P* = 0.005 (mni coordinate = 20, −80, −26, T = 3.10) (Supplementary Figure 1). In contrast, sham stimulation did not result in any significant clusters of activation, even at a lower uncorrected threshold of *P* = 0.005.

**TABLE 3:** Significant findings in the comparisons of tVNS versus sham stimulation.

Regions	MNI coordinate			k	T	*P*-value (FWE-corrected)
	***x***	***y***	***z***			
**tVNS stimulation (concha) > Sham stimulation (earlobe)**						
Superior parietal lobule, left	−32	−58	64	157	4.99	0.010
Precuneus, right	12	−68	52	107	4.28	0.045
Superior parietal lobule, right	48	−40	54	162	4.12	0.008
**Sham stimulation (earlobe) > tVNS stimulation (concha)**						
none						
**1^st^ session: tVNS stimulation (concha) > Sham stimulation (earlobe)**						
None						
**1st session: Sham stimulation (earlobe) > tVNS stimulation (concha)**						
None						
**2nd session: tVNS stimulation (concha) > Sham stimulation (earlobe)**						
Middle frontal gyrus, right	32	20	50	230	4.53	0.001
Superior parietal lobule, right	40	−46	44	156	4.37	0.010
Superior parietal lobule, left	−34	−62	40	195	4.30	0.003
Angular gyrus, right	44	−60	60	106	4.30	0.046
**2nd session: Sham stimulation (earlobe) > tVNS stimulation (concha)**						
None						

MNI, Montreal Neurological Institute; FEW, family-wise error; tVNS, transcutaneous vagal nerve stimulation.

## Discussion

We conducted an fMRI experiment to evaluate the neural correlates of tVNS stimulation in comparison to sham stimulation. Following tVNS, the right frontal gyrus, middle frontal gyrus, right supramarginal gyrus, right precuneus, bilateral superior parietal lobe, left cerebellum, and left lateral occipital cortex exhibited surviving clusters in the fMRI compared to pre-stimulation or sham stimulation fMRI. Additionally, there were no side effects associated with vagal nerve stimulation, such as skin damage, severe pain, fatigue, depressive mood, anxiety, or stress.

These results align with those from a study that employed VNS in epilepsy patients, targeting the frontal, temporal, parietal, and occipital cortices (Sucholeiki et al., 2002). Additionally, a prior study involving twelve healthy individuals subjected to transcutaneous cymba concha stimulation indicated that tVNS is capable of stimulating the brainstem and forebrain. Yakunina et al. (2018) showed that tVNS influenced various frontal and occipital regions in patients with tinnitus.

The stimulated areas in previous studies, as well as in our study, included the fronto-parietal network, also known as the central executive network. The fronto-parietal network includes regions of the prefrontal cortex, cingulate cortex, and posterior parietal cortex, and is associated with executive and cognitive functions and acts as a flexible hub within the brain network for cognitive control (Vincent et al., 2008; Marek and Dosenbach, 2018). This network is involved in sustained attention, novelty detection, problem-solving, working memory, and decision-making (Vincent et al., 2008; Marek and Dosenbach, 2018; De Ridder et al., 2022). Considering the function of the fronto-parietal network, tVNS may have an effect on improving cognitive function and may serve as a treatment strategy for cognitive impairment (Naparstek et al., 2023; Trifilio et al., 2023).

Additionally, our results indicate that tVNS stimulates regions within the fronto-parietal network, which has been extensively studied and associations with depression and tinnitus (Kong et al., 2018; Yakunina and Nam, 2021; Kok et al., 2022; Lee et al., 2022; Zhong et al., 2023). Zhong et al. (2023) demonstrated that depression is associated with decreased functional connectivity of these areas. And some part of the frontoparietal network can be modulated by tVNS, which is associated with the improvement of depression (Kong et al., 2018). In addition, although the increase or decrease of functional connectivity in tinnitus patients is controversial, many previous studies have identified that tinnitus is also associated with aberrant connectivity in the fronto-parietal network (Kok et al., 2022), which is a component of the tinnitus-related triple network and is linked to functional impact (Lee et al., 2022). Yakunina and Nam suggested that tVNS is a potentially therapeutic approach in controlling tinnitus (Yakunina and Nam, 2021) and based on our research, therapeutic effect of tVNS on tinnitus may be attributed from modulation of fronto-parietal network.

The cerebellum and lateral occipital cortex was also stimulated in our study. Cerebellum is connected to the fronto-parietal network, modulating cognitive and executive function (Wang et al., 2023; Zhang et al., 2023). Disruption of this connectivity is associated with some neurological and psychiatric disease such as cognitive impairment or depression (Wang et al., 2023; Zhang et al., 2023). And, lateral occipital cortex involved in the representation and perception of objects and the decreased connectivity of lateral occipital cortex is associated with autism (Jung et al., 2019). Taking into account that previous studies suggest tVNS as a possible treatment strategy for autism, stimulating the lateral occipital cortex with tVNS may be the mechanisms by which tVNS affects autism (Jin and Kong, 2016). Further studies on each disease with stimulated brain regions in strictly controlled situations may be helpful in determining the exact role of tVNS for modulating fronto-parietal network in each condition.

The second session of tVNS revealed clusters that had survived, which were not observed in the first session. These findings suggest that to achieve a sufficient effect from tVNS, at least 3.5 min of stimulation may be necessary. Furthermore, the duration of stimulation could potentially influence the extent of brain area activation. To determine the specific brain areas stimulated in relation to the dosage of tVNS, further research involving additional sessions is required. Moreover, repetitive applications of tVNS with adequate time intervals may produce different outcomes compared to a single session of tVNS. Since brain structural and functional plasticity can be affected by repeated sensory and cognitive stimulation (Teutsch et al., 2008; Biggio et al., 2009; Han et al., 2019; Antonenko et al., 2023), it is possible that recurrent VNS could modify the brain’s structural or functional plasticity. A prospective study focusing on repetitive tVNS stimulation is needed to ascertain the long-term effects of tVNS.

We stimulated for 30-s with 7 blocks for each session, with an average stimulation level of 2.95. However, we did not conduct an fMRI study for varying levels of tVNS stimulation in terms of intensity, frequency of electric stimulation, duration, and number of stimulations. Since the autonomic response and adverse effects vary according to changes in these parameters (Kim et al., 2022; Yokota et al., 2022), it is necessary to identify settings that are more effective with fewer adverse effects. Further study to identify the standard stimulation for tVNS is necessary to provide precise stimulating guidelines.

We used the earlobe as a reference area for tVNS. The earlobe is mainly innervated by the greater auricular nerve, which is a branch of the superficial cervical plexus, originating from the C2 and C3 spinal nerves (Butt et al., 2020). However, some previous studies have identified that stimulating the earlobe can induce functional changes in the brain, sometimes similar to tVNS (Feusner et al., 2012; Yakunina et al., 2017; Butt et al., 2020). In contrast, other previous studies have revealed that stimulating the earlobe is not effective (Butt et al., 2020; de Gurtubay et al., 2021; Kim et al., 2022), and the earlobe has been most commonly used as a reference area (Butt et al., 2020; Kim et al., 2022). Our results also supported that stimulating the earlobe is not as effective as stimulating the cavum conchae and can be used as a reference area. Based on previous studies and our results, the earlobe can be used a valid reference area for sham stimulation.

In our research, we were unable to determine how these changes might influence brain function and structure over time. Recurrent tVNS may prevent functional impairment of the fronto-parietal network (De Ridder et al., 2022) and increase abnormal afferent nodes in fronto-parietal network (Lee et al., 2022), inducing long-term effects similar to results of studies for tVNS in other diseases. Henry et al. (2004) demonstrated that the effects of tVNS could persist for up to 3 months in patients with epilepsy. Similarly, Nahas et al. (2007) reported that changes in brain activity induced by tVNS could last for 5 months in patients with depression. Nonetheless, these time-dependent changes may vary according to the disease status. Further research is needed to evaluate the long-term effects of tVNS on normal population and individuals with each disease, in order to provide clarity on this issue.

A limitation of this study was the small number of subjects. Incorporating results from both the first and second stimulation sessions revealed more surviving clusters than when analyzing each session separately. With a larger sample size, the results might show an even more pronounced survival of clusters. Further study with a greater number of subjects could provide a more precise evaluation of the stimulated region.

In this study, we did not perform tVNS on patients with each neurological or psychiatric diseases. While some researchers have shown that tVNS may be effective for some neurological or psychiatric disease (Kong et al., 2018; Yakunina and Nam, 2021; Vargas-Caballero et al., 2022), the specific brain regions it activates and the appropriate dosage for a beneficial effect without causing skin burns or other side effects associated with vagus nerve stimulation have yet to be determined. Further research involving patients with each neurological or psychiatric disease is required to elucidate the precise mechanisms by which tVNS controls symptoms and the optimal dosage for treatment.

## Conclusion

Here, we show that tVNS stimulation applied to the cavum concha elicits frontal, parietal, and cerebellar activation. These areas are part of the fronto-parietal network, associated with executive and cognitive functions, and have clinical implications. Our results indicate that effect of tVNS on neurological and psychiatric diseases may be mediated through modulation of activity of these brain regions. Furthermore, our findings indicate that a minimum stimulation duration of 3.5 min may be necessary to effectively stimulate these regions.

## Data availability statement

The raw data supporting the conclusions of this article will be made available by the authors, without undue reservation.

## Ethics statement

The study protocol received approval from the Institutional Review Board of Hallym University Sacred Heart Hospital (Anyang, South Korea, IRB No. 2022-08-010-001). The studies were conducted in accordance with the local legislation and institutional requirements. The participants provided their written informed consent to participate in this study.

## Author contributions

S-YH: Writing – original draft, Writing – review and editing, Formal analysis, Methodology. LS: Data curation, Methodology, Writing – review and editing. H-JL: Conceptualization, Formal analysis, Methodology, Project administration, Supervision, Visualization, Writing – review and editing. MP: Conceptualization, Formal analysis, Methodology, Project administration, Resources, Supervision, Writing – review and editing.

## References

[B1] Antonenko D. Fromm A. E. Thams F. Grittner U. Meinzer M. Fløel A. (2023). Microstructural and functional plasticity following repeated brain stimulation during cognitive training in older adults. *Nat. Commun.* 14:3184. 10.1038/s41467-023-38910-x 37268628 PMC10238397

[B2] Berger A. Beckers E. Joris V. Duchìne G. Danthine V. Delinte N. (2024). Locus coeruleus features are linked to vagus nerve stimulation response in drug-resistant epilepsy. *Front. Neurosci.* 18:1296161. 10.3389/fnins.2024.1296161 38469571 PMC10926962

[B3] Biggio F. Gorini G. Utzeri C. Olla P. Marrosu F. Mocchetti I. (2009). Chronic vagus nerve stimulation induces neuronal plasticity in the rat hippocampus. *Int. J. Neuropsychopharmacol.* 12 1209–1221. 10.1017/S1461145709000200 19309534 PMC2879889

[B4] Butt M. F. Albusoda A. Farmer A. D. Aziz Q. (2020). The anatomical basis for transcutaneous auricular vagus nerve stimulation. *J. Anat.* 236 588–611. 31742681 10.1111/joa.13122 PMC7083568

[B5] Chen M. Yu L. Ouyang F. Liu Q. Wang Z. Wang S. (2015). The right side or left side of noninvasive transcutaneous vagus nerve stimulation: based on conventional wisdom or scientific evidence? *Int. J. Cardiol.* 187 44–45. 25828310 10.1016/j.ijcard.2015.03.351

[B6] de Gurtubay I. G. Bermejo P. Lopez M. Larraya I. Librero J. (2021). Evaluation of different vagus nerve stimulation anatomical targets in the ear by vagus evoked potential responses. *Brain Behav.* 11:e2343. 10.1002/brb3.2343 34551214 PMC8613407

[B7] De Ridder D. Vanneste S. Song J. J. Adhia D. (2022). Tinnitus and the triple network model: a perspective. *Clin. Exp. Otorhinolaryngol.* 15 205–212. 10.21053/ceo.2022.00815 35835548 PMC9441510

[B8] Feusner J. D. Madsen S. Moody T. D. Bohon C. Hembacher E. Bookheimer S. Y. (2012). Effects of cranial electrotherapy stimulation on resting state brain activity. *Brain Behav.* 2 211–220. 22741094 10.1002/brb3.45 PMC3381625

[B9] Han J. H. Lee H. J. Kang H. Oh S. H. Lee D. S. (2019). Brain plasticity can predict the cochlear implant outcome in adult-onset deafness. *Front. Hum. Neurosci.* 13:38. 10.3389/fnhum.2019.00038 30837852 PMC6389609

[B10] Henry T. R. Bakay R. A. E. Pennell P. B. Epstein C. M. Votaw J. R. (2004). Brain blood-flow alterations induced by therapeutic vagus nerve stimulation in partial epilepsy: II. prolonged effects at high and low levels of stimulation. *Epilepsia* 45 1064–1070. 10.1111/j.0013-9580.2004.03104.x 15329071

[B11] Howland R. H. (2014). Vagus nerve stimulation. *Curr. Behav. Neurosci. Rep.* 1 64–73. 24834378 10.1007/s40473-014-0010-5 PMC4017164

[B12] Jin Y. Kong J. (2016). Transcutaneous vagus nerve stimulation: a promising method for treatment of autism spectrum disorders. *Front. Neurosci.* 10:609. 10.3389/fnins.2016.00609 28163670 PMC5247460

[B13] Jung M. Tu Y. Lang C. A. Ortiz A. Park J. Jorgenson K. (2019). Decreased structural connectivity and resting-state brain activity in the lateral occipital cortex is associated with social communication deficits in boys with autism spectrum disorder. *Neuroimage* 190 205–212. 10.1016/j.neuroimage.2017.09.031 28927730

[B14] Kim A. Y. Marduy A. De Melo P. S. Gianlorenco A. C. Kim C. K. Choi H. (2022). Safety of transcutaneous auricular vagus nerve stimulation (taVNS): a systematic review and meta-analysis. *Sci. Rep.* 12:22055. 10.1038/s41598-022-25864-1 PMC9772204 36543841

[B15] Kok T. E. Domingo D. Hassan J. Vuong A. Hordacre B. Clark C. (2022). Resting-state networks in tinnitus : a scoping review. *Clin. Neuroradiol.* 32 903–922. 10.1007/s00062-022-01170-1 35556148 PMC9744700

[B16] Kong J. Fang J. Park J. Li S. Rong P. (2018). Treating depression with transcutaneous auricular vagus nerve stimulation: state of the art and future perspectives. *Front. Psychiatry* 9:20. 10.3389/fpsyt.2018.00020 29459836 PMC5807379

[B17] Lee S. J. Park J. Lee S. Y. Koo J. W. Vanneste S. De Ridder D. (2022). Triple network activation causes tinnitus in patients with sudden sensorineural hearing loss: a model-based volume-entropy analysis. *Front. Neurosci.* 16:1028776. 10.3389/fnins.2022.1028776 36466160 PMC9714300

[B18] Lehtimäki J. Hyværinen P. Ylikoski M. Bergholm M. MæKELæ J. P. Aarnisalo A. (2013). Transcutaneous vagus nerve stimulation in tinnitus: a pilot study. *Acta Otolaryngol.* 133 378–382. 23237096 10.3109/00016489.2012.750736

[B19] Marek S. Dosenbach N. U. F. (2018). The frontoparietal network: function, electrophysiology, and importance of individual precision mapping. *Dialogues Clin. Neurosci.* 20 133–140. 30250390 10.31887/DCNS.2018.20.2/smarek PMC6136121

[B20] Nahas Z. Teneback C. Chae J.-H. Mu Q. Molnar C. Kozel F. A. (2007). Serial vagus nerve stimulation functional MRI in treatment-resistant depression. *Neuropsychopharmacology* 32 1649–1660. 10.1038/sj.npp.1301288 17203016

[B21] Naparstek S. Yeh A. K. Mills-Finnerty C. (2023). Transcutaneous Vagus Nerve Stimulation (tVNS) applications in cognitive aging: a review and commentary. *Front. Aging Neurosci.* 15:1145207. 10.3389/fnagi.2023.1145207 37496757 PMC10366452

[B22] Patil J. D. Alrashid M. A. Eltabbakh A. Fredericks S. (2023). The association between stress, emotional states, and tinnitus: a mini-review. *Front. Aging Neurosci.* 15:1131979. 10.3389/fnagi.2023.1131979 37207076 PMC10188965

[B23] Stegeman I. Velde H. M. Robe P. Stokroos R. J. Smit A. L. (2021). Tinnitus treatment by vagus nerve stimulation: a systematic review. *PLoS One* 16:e0247221. 10.1371/journal.pone.0247221 33705401 PMC7951891

[B24] Sucholeiki R. Alsaadi T. M. Morris G. L. Ulmer J. L. Biswal B. Mueller W. M. (2002). fMRI in patients implanted with a vagal nerve stimulator. *Seizure* 11 157–162. 10.1053/seiz.2001.0601 12018958

[B25] Teutsch S. Herken W. Bingel U. Schoell E. May A. (2008). Changes in brain gray matter due to repetitive painful stimulation. *Neuroimage* 42 845–849. 18582579 10.1016/j.neuroimage.2008.05.044

[B26] Trifilio E. Shortell D. Olshan S. O’neal A. Coyne J. Lamb D. (2023). Impact of transcutaneous vagus nerve stimulation on healthy cognitive and brain aging. *Front. Neurosci.* 17:1184051. 10.3389/fnins.2023.1184051 37575296 PMC10416636

[B27] Vargas-Caballero M. Warming H. Walker R. Holmes C. Cruickshank G. Patel B. (2022). Vagus nerve stimulation as a potential therapy in early alzheimer’s disease: a review. *Front. Hum. Neurosci.* 16:866434. 10.3389/fnhum.2022.866434 35572001 PMC9098960

[B28] Ventura-Bort C. Wirkner J. Genheimer H. Wendt J. Hamm A. O. Weymar M. (2018). Effects of transcutaneous vagus nerve stimulation (tVNS) on the P300 and alpha-amylase level: a pilot study. *Front. Hum. Neurosci.* 12:202. 10.3389/fnhum.2018.00202 29977196 PMC6021745

[B29] Ventureyra E. C. (2000). Transcutaneous vagus nerve stimulation for partial onset seizure therapy. a new concept. *Childs Nerv. Syst.* 16 101–102. 10663816 10.1007/s003810050021

[B30] Vincent J. L. Kahn I. Snyder A. Z. Raichle M. E. Buckner R. L. (2008). Evidence for a frontoparietal control system revealed by intrinsic functional connectivity. *J. Neurophysiol.* 100 3328–3342. 18799601 10.1152/jn.90355.2008 PMC2604839

[B31] Wang X. Xia J. Wang W. Lu J. Liu Q. Fan J. (2023). Disrupted functional connectivity of the cerebellum with default mode and frontoparietal networks in young adults with major depressive disorder. *Psychiatry Res.* 324:115192. 10.1016/j.psychres.2023.115192 37054552

[B32] Yakunina N. Kim S. S. Nam E. C. (2017). Optimization of transcutaneous vagus nerve stimulation using functional MRI. *Neuromodulation* 20 290–300. 27898202 10.1111/ner.12541

[B33] Yakunina N. Kim S. S. Nam E. C. (2018). BOLD fMRI effects of transcutaneous vagus nerve stimulation in patients with chronic tinnitus. *PLoS One* 13:e0207281. 10.1371/journal.pone.0207281 30485375 PMC6261575

[B34] Yakunina N. Nam E.-C. (2021). Direct and transcutaneous vagus nerve stimulation for treatment of tinnitus: a scoping review. *Front. Neurosci.* 15:680590. 10.3389/fnins.2021.680590 34122002 PMC8193498

[B35] Yap J. Y. Y. Keatch C. Lambert E. Woods W. Stoddart P. R. Kameneva T. (2020). Critical review of transcutaneous vagus nerve stimulation: challenges for translation to clinical practice. *Front. Neurosci.* 14:284. 10.3389/fnins.2020.00284 32410932 PMC7199464

[B36] Yokota H. Edama M. Hirabayashi R. Sekine C. Otsuru N. Saito K. (2022). Effects of stimulus frequency, intensity, and sex on the autonomic response to transcutaneous vagus nerve stimulation. *Brain Sci.* 12:1038. 10.3390/brainsci12081038 36009101 PMC9405815

[B37] Zhang P. Duan L. Ou Y. Ling Q. Cao L. Qian H. (2023). The cerebellum and cognitive neural networks. *Front. Hum. Neurosci.* 17:1197459. 10.3389/fnhum.2023.1197459 37576472 PMC10416251

[B38] Zhong J. Xu J. Wang Z. Yang H. Li J. Yu H. (2023). Changes in brain functional networks in remitted major depressive disorder: a six-month follow-up study. *BMC Psychiatry* 23:628. 10.1186/s12888-023-05082-3 37641013 PMC10464087

