# Supplementary figures and images for: Transcutaneous auricular vagus nerve stimulation can modulate fronto-parietal brain networks
Source: Front Neurosci. 2024 Jul 18;18:1368754. doi: 10.3389/fnins.2024.1368754 (PMC11292796; doi:10.3389/fnins.2024.1368754)

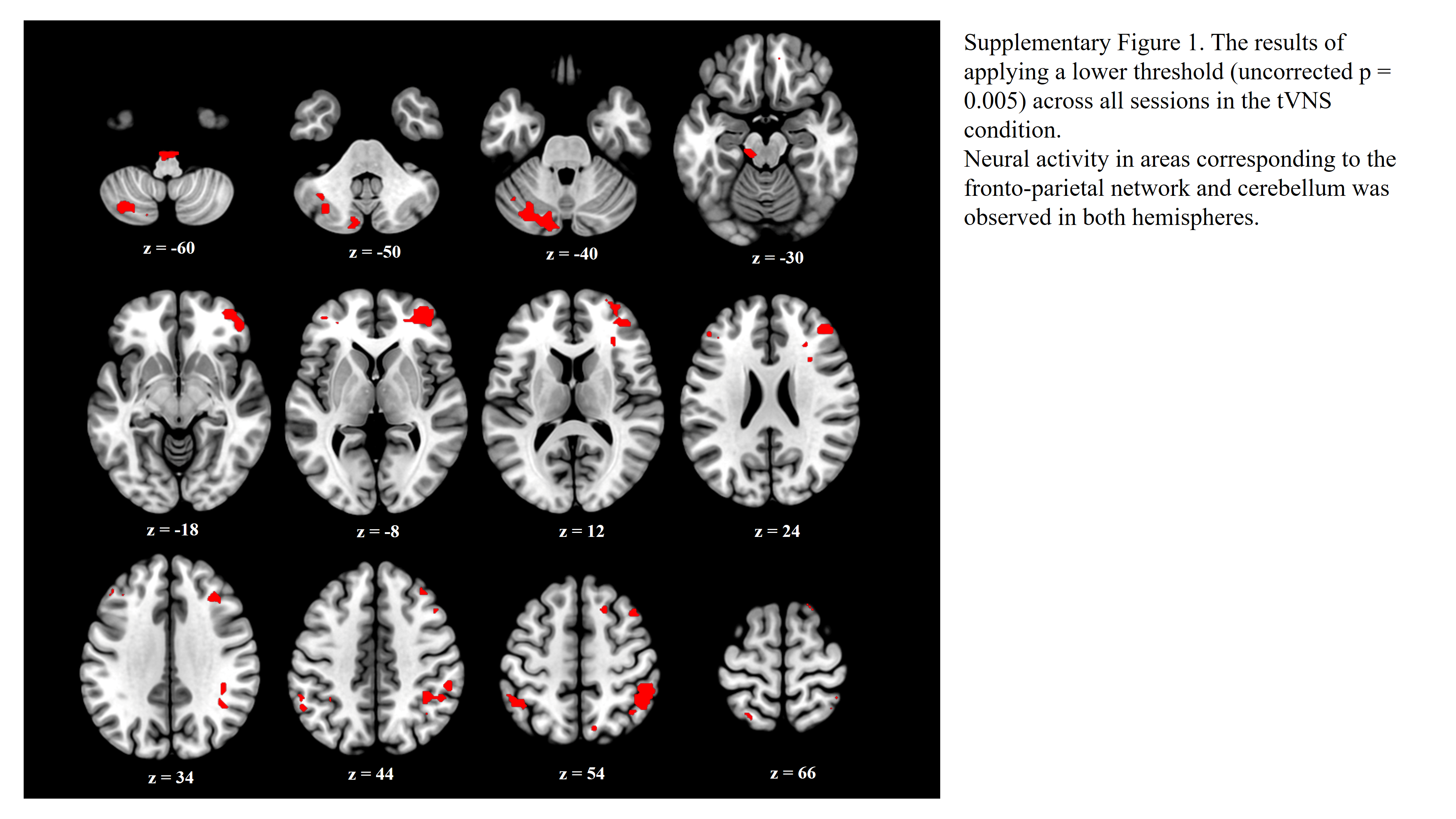

Supplement: Supplementary file 1 [file Image_1.TIF]
